# NAVIGATING THE DIGITAL DIVIDE: FACTORS INFLUENCING PATIENT PORTAL ADOPTION BY OLDER ADULTS DURING THE PANDEMIC

**DOI:** 10.1093/geroni/igad104.3489

**Published:** 2023-12-21

**Authors:** Chung Hyeon Jeong, BoRin Kim

**Affiliations:** University of New Hampshire, Durham, New Hampshire, United States; University of New Hampshire, Durham, New Hampshire, United States

## Abstract

The pandemic rapidly accelerated the adoption of digital healthcare technologies, including patient portals. The patient portals enhance patient-provider communication, streamline medical records management, and provides tailored education materials. Nevertheless, older patients, known as heavy healthcare users, often encounter challenges when adopting patient portals. This digital divide could lead to worsening health and healthcare disparities. Guided by the Senior Technology Acceptance and Adoption Model (STAM), this study explored the factors influencing the adoption of patient portals by older adults during the pandemic. We conducted semi-structured virtual interviews in 2021 with 31 older adults aged 60 or older with healthcare needs. Interviews were thematically coded and analyzed using constant comparative methods. The STAM proposes an orderly phase of technology acceptance among older adults, comprising three phases: 1) objectification, 2) incorporation, and 3) conversion. Our findings showed that some older patients felt compelled to use patient portals during the pandemic, bypassing the objectification phase, which is crucial for building an intention to use the new technology. Older patients who lack a strong intention to use patient portals tended to report challenges in the incorporation phase, rather than focusing on the usefulness of patient portals. From the interviews, we identified three overarching themes: 1) perceived concerns or limited benefits, 2) aging-related and system-related barriers, and 3) limited training opportunities. These themes could pose obstacles to the adoption of patient portals during the conversion phase. The findings of this study suggest areas for improvement to increase the adoption of patient portals among older patients.

